# CRISPR applications in cancer diagnosis and treatment

**DOI:** 10.1186/s11658-023-00483-4

**Published:** 2023-09-06

**Authors:** Mingxia Wang, Menghui Chen, Xia Wu, Xinbo Huang, Bo Yu

**Affiliations:** 1 Department of Dermatology, Skin Research Institute of Peking University Shenzhen Hospital, Peking University Shenzhen Hospital, Shenzhen, 518036 China; 2 Shenzhen Key Laboratory of Reproductive Medicine and Genetics, Institute of Urology, Peking University Shenzhen Hospital, Shenzhen, 518000 China

**Keywords:** Cas9, Cas12, Cas13, Cancer, Gene therapy, Diagnostic tools, Clinical trials

## Abstract

Cancer remains a significant global health challenge, necessitating the exploration of novel and more precise therapeutic options beyond conventional treatments. In this regard, clustered regularly interspaced short palindromic repeats (CRISPR) systems have emerged as highly promising tools for clinical gene editing applications. The CRISPR family encompasses diverse CRISPR-associated (Cas) proteins that possess the ability to recognize specific target sequences. The initial CRISPR system consisted of the Cas9 protein and a single-guide RNA, which guide Cas9 to the desired target sequence, facilitating precise double-stranded cleavage. In addition to the traditional cis-cleavage activity, the more recently discovered Cas12 and Cas13 proteins exhibit trans-cleavage activity, which expands their potential applications in cancer diagnosis. In this review, we provide an overview of the functional characteristics of Cas9, Cas12, and Cas13. Furthermore, we highlight the latest advancements and applications of these CRISPR systems in cancer gene therapy and molecular diagnosis. We also emphasize the importance of understanding the strengths and limitations of each CRISPR system to maximize their clinical utility. By providing a comprehensive overview of the current state of CRISPR technology in cancer research, we aim to inspire further exploration and innovation in this rapidly evolving field.

**Graphical abstract:**

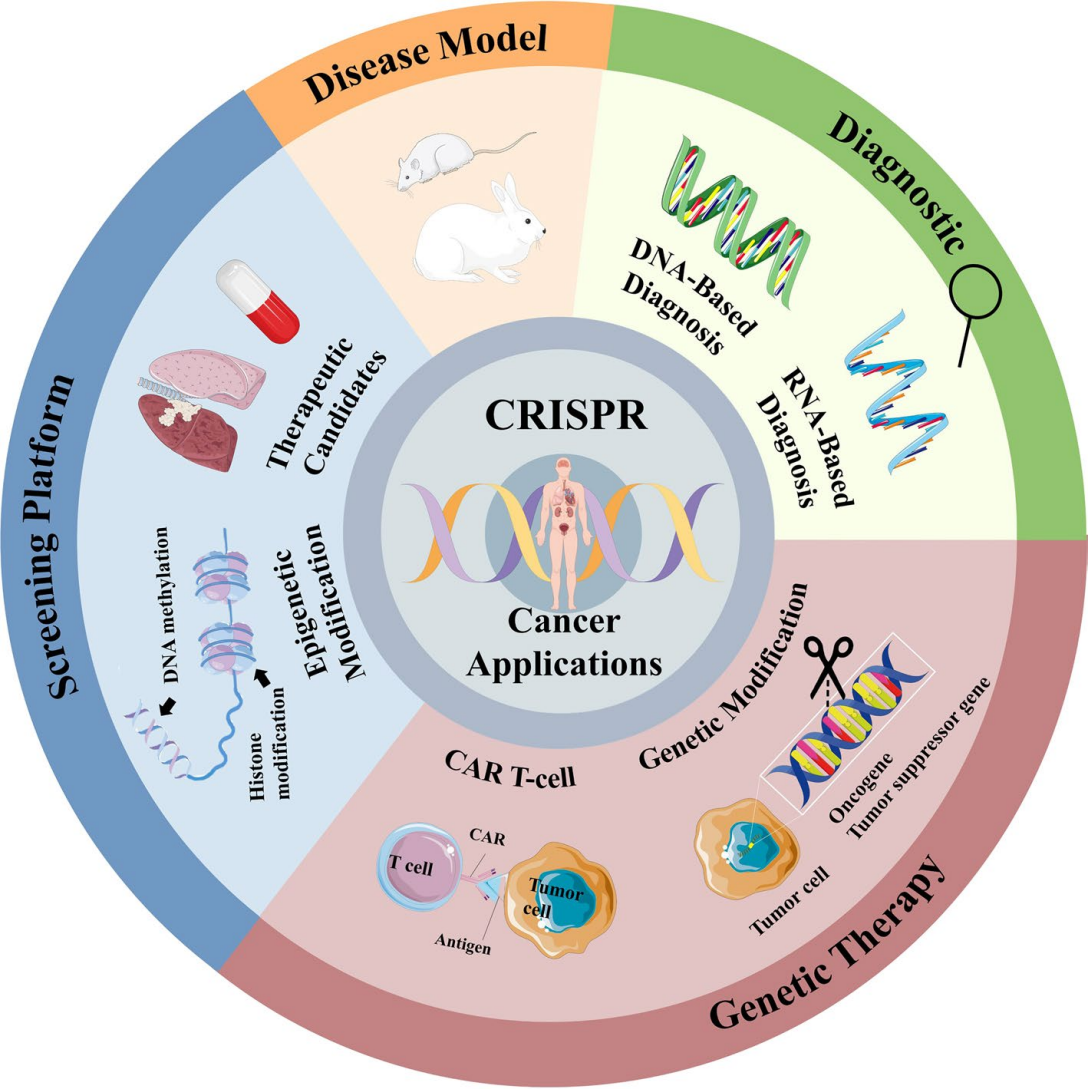

## Introduction

Cancer, characterized by an unrestrained and invasive proliferation of abnormal cells [1], has long been the leading cause of death worldwide and remained a vital public health concern [2] (https://gco.iarc.fr/today/fact-sheets-cancers). Despite being the cornerstone of cancer treatment, traditional therapies such as surgery [3], chemotherapy [4], and radiation therapy [5] have limitations including lack of specificity, significant side effects, and challenges in preventing disease progression or recurrence [6]. Therefore, there is an urgent need to develop therapeutic strategies that offer enhanced precision, effectiveness, and tolerability. One such advancement is gene therapy, which involves manipulating genetic material to correct cancer-causing genetic mutations [7]. With the growing understanding of cancer genomics, gene editing technologies like CRISPR are revolutionizing the potential for precision cancer medicine [8].

The discovery of clustered regularly interspaced palindromic repeats (CRISPR) in Escherichia coli in 1987 laid the foundation for understanding its functional role in bacteria [9], which was elucidated in 2003 when Mojica suggested their contribution to microbial adaptive immunity [10]. The turning point in the field occurred when the first CRISPR-associated protein (Cas), Cas9, was used to cleave the DNA in vitro in 2012 [11, 12]. Following this groundbreaking discovery, researchers demonstrated its programmable ability and showcased its application in editing eukaryotic genes both in vitro and in vivo [13–15].

CRISPR-Cas systems are classified into two major classes [16]. Class 1 systems, found primarily in bacteria and archaea, constitute approximately 90% of known CRISPR-Cas loci and are composed of heteromeric multiprotein effectors [17]. Class 2 systems, accounting for about 10% of CRISPR-Cas loci, are more frequently used due to their single multidomain effector, which is easier to manipulate [18]. Class 2 CRISPR-Cas systems can be divided into three types (type II, V, and VI), each with distinct properties and mechanisms [19]. Type II, exemplified by Cas9, is the most well-known and extensively studied system, responsible for double-stranded DNA cleavage [19]. Type V, represented by Cas12, also targets DNA, while type VI, represented by Cas13, has a different structure and targets single-stranded RNA (ssRNA) [20, 21].

Among CRISPR-based editing systems, CRISPR-Cas9 has been extensively investigated both in vitro and in vivo. Over the years, researchers have made various modifications to improve its specificity and efficiency, leading to promising preliminary results in ongoing clinical trials [22]. Recently discovered Cas12 and Cas13 systems have also shown potential in gene therapy, with the additional advantage of collateral cleavage activity, which enables their use as sensitive and specific diagnostic tools [23, 24].

This review provides a comprehensive summary of the structural and functional characteristics of signature Class 2 Cas proteins, including Cas9, Cas12, and Cas13, as well as their applications in cancer gene therapy and molecular diagnosis. The inclusion of up-to-date research and clinical trials highlights the current progress in the field, while also addressing the key limitations of different CRISPR tools. Although CRISPR-based therapies and diagnostics offer great promise, further rigorous trials and long-term follow-up are needed to establish their safety and efficacy.

### DNA editing mechanism of CRISPR-Cas9

**Fig. 1:**
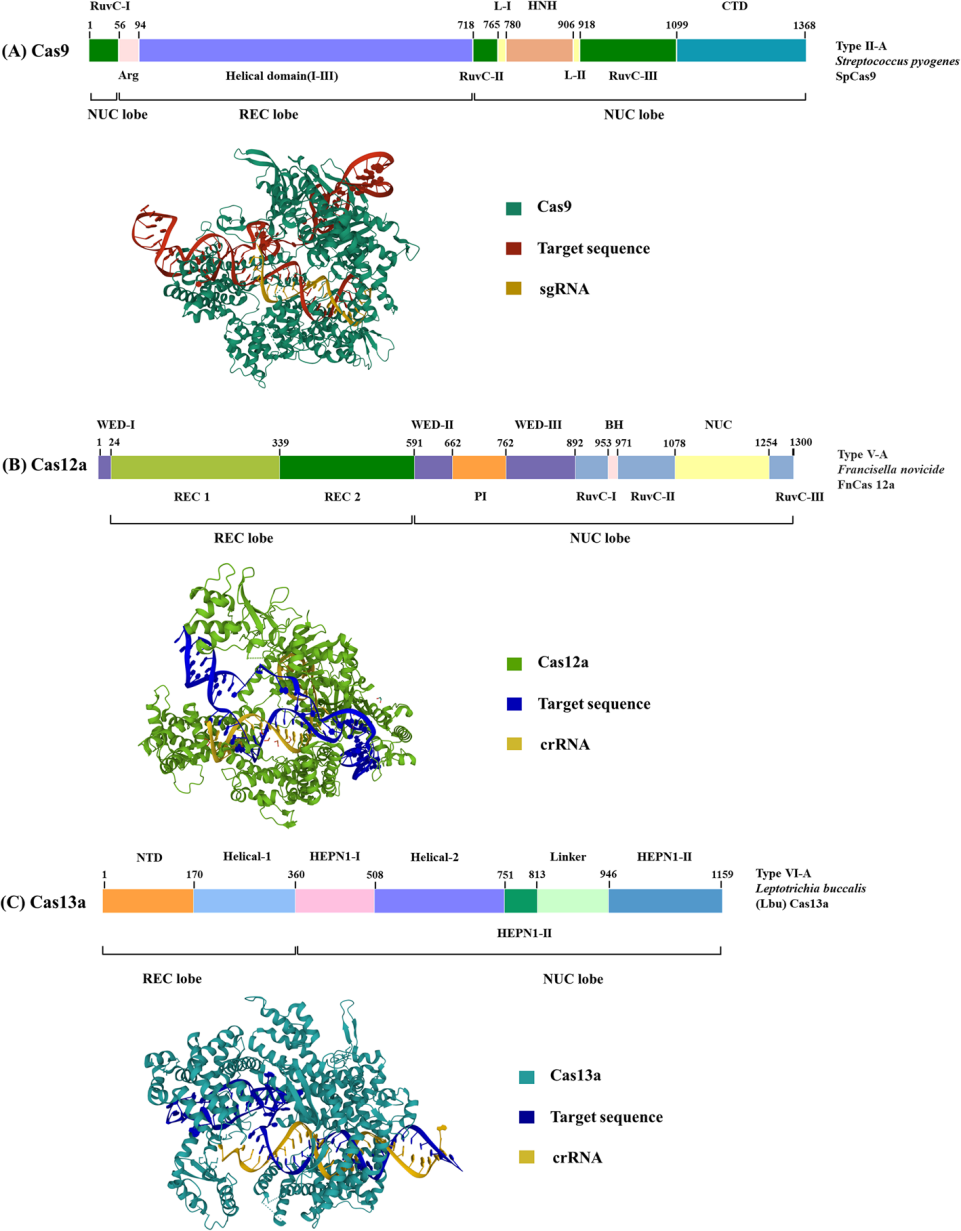
Schematics of the domain organization of Cas proteins and crystal structures of Cas protein-sgRNA-target sequence complex. **A** SpCas9 (PDB: 4OO8) (**B**) FnCas12a (PDB: 6I1K) (**C**) LbuCas13a (PDB: 5WXP). *REC* recognition lobe, *NUC* nuclease lobe, *BH* bridge helix, *Arg* arginine-rich bridge helix, *CTD* C-terminal domain, *WED* Wedge, *PI* protospacer adjacent motif (PAM) interacting, *NTD* N-terminal domain, *HEPN* higher eukaryotes and prokaryotes nucleotide-binding domains

**Fig. 2:**
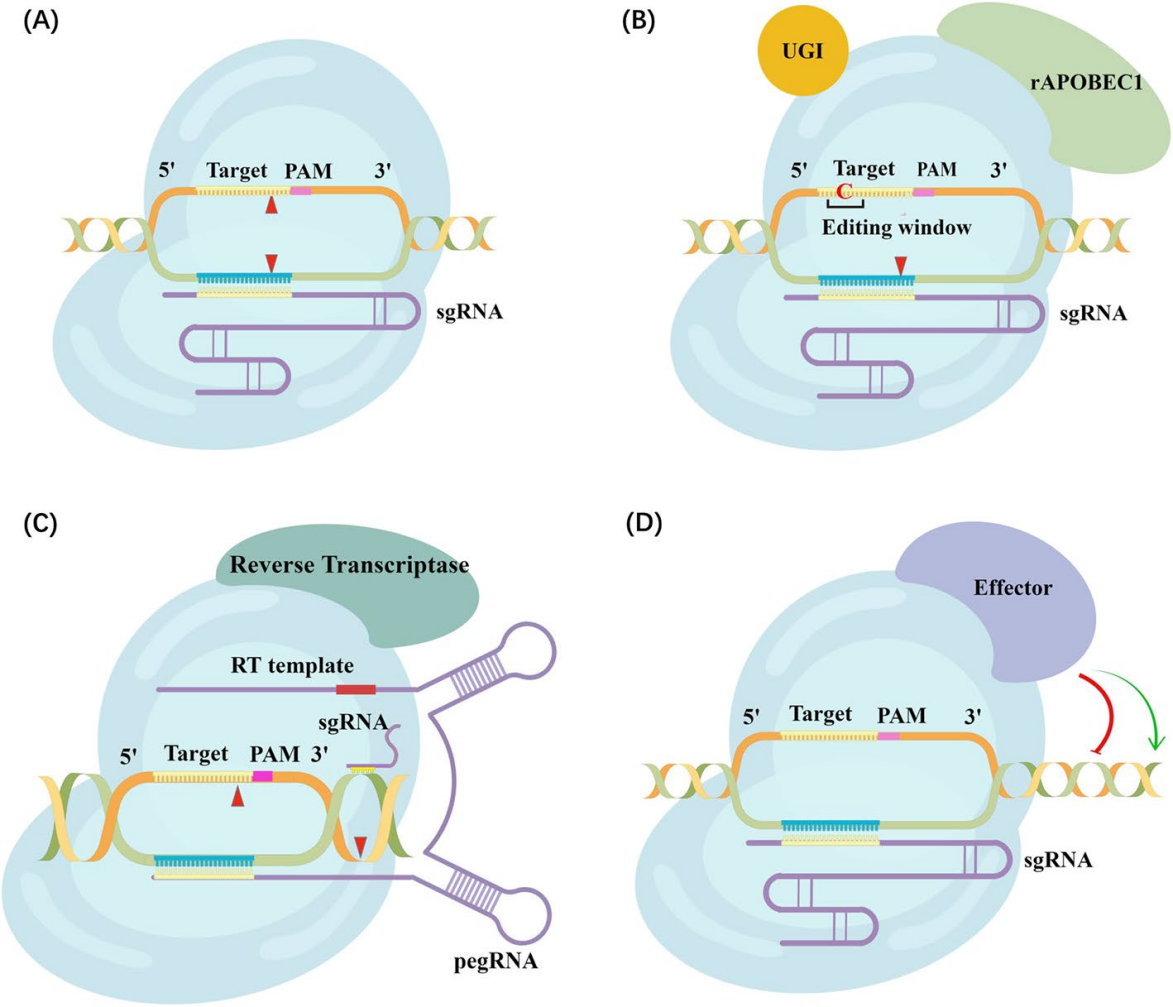
Schematic of various CRISPR-Cas9 functions. **A** Classical CRISPR/Cas9 works as genetic scissors for a specific edition. The Cas9 nuclease (light blue) recognizes 3′ PAM sequence (NGG, NAG) (pink) and target DNA pairs with complementary sgRNA (purple), followed by the formation of blunt DNA double-stranded breaks at the target site (red). Cas9 was fused with selected proteins to induce distinct biological effects (**B**–**D**). **B** dCas9 protein fuses to the cytosine deaminase APOBEC1 (light green) and the uracil DNA glycosylase inhibitor (UGI, orange) to form cytosine base editor. The dCas9 component enables the guidance to its target sequence, then the APOBEC1 component induces C to T conversion. **C** Cas9 nickase fuse with reverse transcriptase (RT, green) to form prime editors. The prime-editing guide RNA (pegRNA, purple) binds to its complementary target DNA, and the unbound DNA of the PAM-containing strand is cleaved by Cas9. This creates a complementary primer for the desired sequence on the pegRNA, and the RT actively extended the unbound DNA using the pegRNA as a template. **D** dCas9 fuses to transcriptional activator or repressor (purple) to create CRISPRa and CRISPRi tools and enable target gene transcription regulation

CRISPR-Cas9 is a genome editing system composed of two essential components: guide RNA (gRNA) and Cas9. The Cas9 protein exhibits a bi-lobed structure, comprising the recognition lobe (REC) with three Helical domains and a Bridge Helix, and the nuclease lobe (NUC) containing a split RuvC domain, an HNH domain, a Topo domain, and a C-terminal domain (CTD) (Fig. 1A). These structural elements play crucial roles in target recognition, DNA binding, and cleavage, guided by the sequence of the gRNA [12]. The gRNA is artificially synthesized by combining CRISPR RNA (crRNA) and trans-activating crRNA (tracrRNA), where the crRNA identifies the DNA target while the tracrRNA binds to the Cas9 protein [12]. The editing process initiates with Cas9 recognizing the protospacer adjacent motif (PAM) sequence, typically 5'-NGG-3' (N representing any nucleotide) [25]. The gRNA then pairs with the complementary target sequence [26, 27], leading to the activation of the RuvC and HNH domains of the Cas9, resulting in the cleavage of the non-target and target DNA strands, respectively [28]. This generates a blunt double-stranded break (DSB) at three base pairs upstream of PAM site (Fig. 2A).

Upon DSB formation, DNA repair mechanisms are triggered, primarily via two pathways: homology-directed repair (HDR) and nonhomologous end joining (NHEJ) [29]. HDR requires a homologous DNA template and is most efficient during the late S and G2 phases of the cell cycle [30]. It allows the introduction of specific point mutations or the insertion of desired sequences through DNA template recombination [31]. Although this pathway is preferred for restoring cellular function, it occurs less frequently than NHEJ. NHEJ operates throughout the cell cycle and repairs DSBs by directly joining DNA fragments without the need for external homologous DNA [30]. Despite its higher occurrence rate, it often results in small, random insertions or deletions that cause frameshift mutations or premature stop codons [32–34].

Having elucidated the mechanism of CRISPR-Cas9, researchers have explored its diverse applications across various fields, including disease model generation, gene therapy, and high-throughput screening platforms for comprehensive gene analysis [35]. Additionally, numerous Cas9-based therapeutics are currently undergoing clinical trials, showcasing promising results and highlighting the immense potential for future gene therapy endeavors.

### Application of CRISPR-Cas9 in cancer modelling

The CRISPR-Cas9 system has played a pivotal role in the development of disease models, particularly for cancer and genetic diseases [36, 37]. For instance, Platt et al*.* established a Cas9 knock-in mouse model by delivering the CRISPR system to the lung via adeno-associated virus (AAV). They established adenocarcinoma pathology through targeted mutations in frequently altered genes such as Tumor Protein P53 (TP53), Liver kinase B1 (LKB1), or Kirsten ratsarcoma viral oncogene homolog (KRAS) [38].

In addition to mouse models, the advent of organoids has expanded the scope of gene therapy research. Lo et al*.* used CRISPR/Cas9 to generate a knockout (KO) of the tumor suppressor gene AT-rich interaction domain 1A (ARID1A) in primary TP53(−/−) human gastric organoids. This led to morphologic dysplasia, tumorigenicity, and mucinous differentiation [39]. Zhao et al*.* reported the generation of a Cas9-edited TP53 and cyclin-dependent kinase inhibitor 2A (CDKN2A) KO gastro-esophageal junction (GEJ) organoid model. This study revealed the important role of TP53/CDKN2A inactivation in early GEJ neoplasia, which may aid in early diagnosis and prevention [40]. The efficient and straightforward nature of CRISPR-Cas9 as a method for creating disease models has significantly advanced our understanding of disease pathologies.

### Functional gene screening and epigenetic regulation in cancer cells via CRISPR-Cas9

Moreover, the CRISPR-Cas9 system has revolutionized high-throughput screening of gene activities in various biological processes, including tumor growth, metastasis, therapeutic resistance, and response to immunotherapy [41, 42]. For example, Shalem et al*.* developed a genome-scale CRISPR-Cas9 knockout (GeCKO) library and identified that numerous genes, both previously validated and newly discovered, that contribute to vemurafenib resistance in a melanoma model [43]. Similarly, Wei et al*.* utilized the CRISPR-Cas9 screening tool to identify phosphoglycerate dehydrogenase (PHGDH) as a driver of resistance in hepatocellular carcinoma (HCC) treatment. The use of the PHGDH inhibitor NCT-503 successfully overcame the resistance, resulting in the abolition of in vivo HCC growth [44]. Additionally, Chen et al*.* identified Haspin (GSG2) as targetable kinases that displayed synthetic lethal interactions with selective Aurora-A inhibitor alisertib (MLN8237) in breast cancer cells. They also discovered the Haspin inhibitor CHR-6494, which synergistically reduced tumor growth, enhancing the therapeutic effects of MLN8237 in a combinational therapy [45]. These findings underscore the significant impact of the CRISPR-Cas9 screening platform in expediting new drug development and benefiting the pharmaceutical industry.

In addition to its role in gene editing, extensive research has explored the transcriptional regulatory capabilities of CRISPR-Cas9. Qi et al*.* first introduced the concept of dCas9, which refers to an endonuclease-deficient mutant Cas9 that retains its target recognition capability but lacks nuclease activity [46]. By fusing dCas9 with transcriptional regulators, the CRISPR activation (CRISPRa) and interference (CRISPRi) tools were developed [47–49]. These tools enable the epigenetic regulation of target gene expression (Fig. 2D). Building upon these tools, Cui et al*.* established a dual CRISPR interference and activation (CRISPRi/a) system that simultaneously silenced the X-inactive specific transcript (XIST) and activated Forkhead box P3 (FOXP3) in breast cancer cells. This manipulation led to increased H4 acetylation and decreased DNA methylation, highlighting the potential of CRISPRi/a for transcriptional and epigenetic modifications [50].

The regulatory capacity of CRISPR-Cas9 has sparked the development of screening platforms based on CRISPRa and CRISPRi for functional studies [51–53]. For example, Myacheva et al*.* utilized this screening strategy in human lung adenocarcinoma (LUAD) and identified CASP8AP2 as a key regulator influencing the viability of all eight examined LUAD cell lines [54]. Another research group conducted a super enhancer screening in leukemic cells, revealing the specific expression of receptors associated with cell growth and survival in acute leukemia [55]. The development of dCas9 and its fusion with regulatory domains holds great promise for precise regulation of target gene expression. Furthermore, CRISPRa and CRISPRi screening platforms offer significant opportunities for genome-wide functional studies.

### Enhancing cancer therapy through CAR-T cell engineering by CRISPR-Cas9

CRISPR-Cas9 has been extensively investigated for its potential in gene therapy, particularly in the context of cancer treatment, which presents complex challenges and high mortality rates. The application of CRISPR-Cas9-mediated knock-out or knock-in strategies offers a valuable approach to interfere with oncogene expression and evaluate its impact on tumor progression [41]. Encouraging results have been obtained from clinical trials employing CRISPR-Cas9 in patients with refractory cancer, particularly in the realm of T cell therapy, where synthetic chimeric antigen receptor (CAR)-T cells have been programmed using CRISPR-Cas9 [56]. CAR-T cells are genetically engineered to specifically target tumor antigen-expressing cells, independent of the major histocompatibility complex (MHC), which is often implicated in immune evasion by tumors. While the production of individual autologous CAR-T cells is costly and complex, the simplicity of CRISPR-Cas9 technology allows for the development of allogeneic CAR-T cells, including T-cell receptor β (TCRβ), programmed cell death protein 1 (PD-1), cytotoxic T lymphocyte-associated antigen-4 (CTLA-4), and more [57, 58]. These modified T cells have demonstrated both in vitro and in vivo efficacy without causing graft-versus-host disease (GVHD), a major complication of transplantation therapy [57, 58].

Recent clinical trials utilizing CAR-T cells have shown promising outcomes in cancer therapy. For instance, You et al*.* reported improvements in median progression-free survival and median overall survival in advanced non-small-cell lung cancer (NSCLC) patients treated with PD-1 edited T cells (NCT02793856) (Table 1) [59]. Other groups have targeted multiple genes in T cell. Stadtmauer et al*.* used CRISPR-Cas9 to remove the endogenous TCRα, TCRβ, and the PD-1 from CAR-T cells specific to New York esophageal squamous cell carcinoma 1(NY-ESO-1). This approach successfully reduced TCR mispairing and improved antitumor immunity [60]. Zhang et al*.* administered anti-CD19 CAR-T cells with integrated PD-1 into patients with relapsed/refractory aggressive B cell non-Hodgkin lymphoma (NCT04213469) (Table 1). The researchers reported a substantial rate of complete remission (87.5%) and long-term response, without any serious adverse events [61]. In addition, Hu et al*.* infused CRISPR-edited CD19/CD22 dual-targeted CAR-T cells to six patients with relapsed/refractory acute lymphoblastic leukemia, achieving a relatively high complete remission rate without genotoxicity or chromosomal translocation [62]. Trial focusing on solid tumours have also been conducted, Foy et al*.* designed transgenic T cells with knockout of the endogenous TCR α constant (TRAC) and TCR β constant (TRBC), and the insertion of two chains of a patient-specific neoantigen-specific TCR (neoTCR) into the TRAC locus. Five out of the sixteen participants demonstrated stable disease, while the others experienced disease progression [63]. These clinical trials of CRISPR-Cas9 have laid a solid foundation for the future widespread clinical application of gene therapy.

**Table 1:** Summarization of ongoing CRISPR related clinical trials

Classification	Identifier	Targeted disease	Targeted gene	Start date	Intervention/treatment	Estimated enrollment	Phase
CRISPR/Cas9	NCT02793856	Metastatic non-small cell lung cancer	Programmed cell death protein 1(PDCD1) gene	August 28, 2016	Autologous CRISPR Cas9 mediated PD-1 Knockout T cells	12	Phase 1
	NCT03044743	Advanced Stage EBV Associated Malignancies	PD-1	April 7, 2017	CRISPR-Cas9 mediated PD-1 knockout-T cells from autologous origin	20	Phase 1/2
	NCT03164135	HIV-infected Subjects With Hematological Malignances	CCR5	May 30, 2017	CD34 + hematopoietic stem/progenitor cells from donor are treated with CRISPR/Cas9 targeting CCR5 gene	5	–
	NCT03166878	Relapsed or Refractory CD19 + Leukemia and Lymphoma	CD19	June 2017	Universal CRISPR-Cas9 Gene-Editing CAR-T Cells Targeting CD19	80	Phase 1/2
	NCT03398967	Relapsed or Refractory B Cell Leukemia and Lymphoma	CD19 and CD20 or CD22	January 2, 2018	Universal CRISPR-Cas9 Gene-Editing CAR-T Cells Targeting CD19 and CD20 or CD22	80	Phase 1/2
	NCT03545815	Mesothelin Positive Multiple Solid Tumors	PD-1 and TCR	March 19, 2018	CRISPR-Cas9 Mediated PD-1 and TCR Gene-knocked Out Mesothelin-directed CAR-T Cells	10	Phase 1
	NCT03747965	Mesothelin Positive Multiple Solid Tumors	PD-1	November 2018	CRISPR-Cas9 Mediated PD-1 Gene-knocked Out Mesothelin-directed CAR-T Cells	10	Phase 1
	NCT04417764	Advanced Hepatocellular Carcinoma	PD-1	June 20, 2019	Autologous PD-1 Knockout CRISPR-Cas9-Engineered T Cells	10	Phase 1
	NCT04035434	Relapsed or Refractory B-Cell Malignancies	CD19	July 22, 2019	CD19-directed Allogeneic CRISPR-Cas9-Engineered T Cells (CTX110)	143	Phase 1
	NCT04244656	Relapsed or Refractory Multiple Myeloma	BCMA (B-cell maturation antigen)	January 22, 2020	Anti-BCMA(B-cell maturation antigen) Allogeneic CRISPR-Cas9-Engineered T Cells (CTX120)	80	Phase 1
	NCT04213469	Relapse/Refractory B-cell Lymphoma	PD1 and CD19	March 13, 2020	PD1 specific integrated anti-CD19 Chimeric Antigen Receptor T Cells	20	–
	NCT04426669	Metastatic Gastrointestinal Epithelial Cancer	CISH (Cytokine-induced SH2 protein)	May 15, 2020	Autologous CISH (Cytokine-induced SH2 protein) inactivated CRISPR-Cas9-Engineered Tumor-Infiltrating Lymphocytes	20	Phase 1/2
	NCT04438083	Relapsed or Refractory Renal Cell Carcinoma With Clear Cell Differentiation	CD70	June 16, 2020	CD70-directed Allogeneic CRISPR-Cas9-Engineered T Cells (CTX130)	107	Phase 1
	NCT04502446	Relapsed or Refractory T or B Cell Malignancies	CD70	July 31, 2020	Anti-CD70 Allogeneic CRISPR-Cas9-Engineered T Cells (CTX130)	45	Phase 1
CRISPR/Cas12	NCT05447169	Nasopharyngeal Carcinoma	Epstein-Barr virus DNA	July 10, 2022	Quantitative polymerase chain reaction and Cas 12a target sequencing of EBV DNA in nasopharyngeal brushing and plasma	11,625	–

### Off-target editing of CRISPR-Cas9

While CRISPR-Cas9 editing holds great potential for clinical research, it faces challenges that impact its specificity and efficiency. Alanis-Lobato et al*.* observed off-target editing in approximately 16% of human embryonic cells [58], while Boutin et al*.* detected megabase-scale losses of heterozygosity (LOH) [64]. Liu et al*.* found unwanted repairing byproducts such as deletions, vector integrations, and chromosomal translocations [65], and Tuladhar et al*.* noticed the production of functional foreign mRNAs and aberrant proteins in approximately 50% of CRISPR-Cas9 edited cell lines [66]. Until these fundamental issues are resolved, the use of CRISPR-Cas9 in gene therapy remains limited in terms of safety and feasibility.

To address some of the limitations of CRISPR-Cas9 editing, novel CRISPR-based tools such as base editors and prime editors have been developed. These tools aim to avoid the induction of double-stranded breaks (DSBs) and the risk of deletions, insertions, and frameshift mutations during DNA repair processes [67]. Base editors enable highly efficient and precise single-base substitutions [68, 69]. Prime editors allow the induction of transitions, transversions, small insertions, and deletions without the need for an exogenous donor template [70]. These tools allow for more specific donor-free editing in a programmable manner.

### Expanding therapeutic opportunities with novel CRISPR tools

The first CRISPR-based base editor was developed by Komor et al*.* in 2016 [68]. They fused the cytosine deaminase Apolipoprotein B MRNA Editing Enzyme Catalytic Subunit 1 (APOBEC1) to the amino terminus of dead Cas9 (dCas9). The dCas9 component enables target sequence guidance, while the APOBEC1 component induces C to T conversion (Fig. 2B) [68]. Subsequently, Adenine base editors inducing A to G conversions [69], and C-to-G base editors (CGBE) inducing C-to-G base transversions[71] were developed. Numerous enhanced base editors with improved efficiency and specificity have since been developed [68, 72–74]. In 2021, Wang et al*.* constructed a new transformer base editing (tBE) system that increases on-target specificity while reducing unintended mutations [75]. Later, Zhang et al*.* developed a dual adenine and cytosine base editor system by combining both deaminases with a Cas9 nickase, thereby expanding the scope of base editors to enable C-to-T and A-to-G conversions at the same target [74]. Though research efforts have significantly improved the efficiency, targeting scope, and product purity of base editors [67, 76], drawbacks including limitations in targetable sites and unanticipated off-target editing hinder their feasibility in therapeutic use.

Base editors have proven to be highly valuable in large-scale genome screening to identify gain- and loss-of-function variants [77, 78], as well as pathogenic variants of cancer [79]. These applications enhance our understanding of the functional implications of cancer-associated variants. The screening system has also been used to investigate the impact of protein modifications, such as phosphorylation, on chemotherapy response. For instance, Li et al*.* revealed the involvement of P21-activated Kinase 4/Extracellular Regulated Protein Kinases (PAK4/ERK) and Ribosomal S6 Kinase 2/Recombinant Tumor Protein p53 Binding Protein 1/ Phosphorylated H2A Histone family member X (RSK2/TP53BP1/γ-H2AX) signaling pathways in anti-chemoresistance [80].

Base editors have shown significant therapeutic potential in directly modifying oncogenes or tumor suppressor genes. Annunziato et al*.* employed CBEs to modify oncogenic mutations in a triple-negative breast cancer model [81]. Sayed et al*.* used ABEs to correct KRAS and TP53 mutations in cancer organoids from patients [82]. Both studies provided proof of concept for extending the use of flexible base editing tools to other tumor types. Additionally, base editors have been utilized in the development of CAR-T platforms, demonstrating the disruption of multiple genes with high efficiency and minimal double-strand breaks [83]. Diorio et al*.* achieved promising results by developing a CD7-directed allogeneic CAR-T (7CAR8) using CBEs, which showed high effectiveness against T-cell acute lymphoblastic leukemia (T-ALL) in various in vitro and in vivo models [84]. These advancements hold significant translational potential for cancer treatment.

Another breakthrough in precise gene editing is prime editing, which incorporates an engineered reverse transcriptase (RT) with Cas9 nickase [70]. Together with a prime-editing guide RNA (pegRNA), they can precisely modify target sequences in a programmable manner. The pegRNA specifically binds to the complementary target DNA, and Cas9 cleaves the unbound DNA of the PAM-containing strand. This generates a complementary primer for the desired sequence on the pegRNA, and the RT actively extends the unbound DNA utilizing the pegRNA as a template. The resulting edited 3' flap aims to integrate into the genome while the original 5' flap is vulnerable to cellular endonucleases during lagging-strand DNA synthesis (Fig. 2C) [70].

Prime editing represents a revolutionary advancement in precise gene editing. The initial version, Prime Editor 1 (PE1), integrated features of both CRISPR and reverse transcriptase but displayed suboptimal efficiency for mass application [70]. Subsequent versions, Prime Editor 2 (PE2) and Prime Editor 3 (PE3), showed steady progress, with PE2 improving editing efficacy by merging a designed reverse transcriptase with Cas9 nickase, and PE3 further enhancing efficiency by inducing strand replacement [85]. However, prime editing has certain limitations compared to other technologies such as CRISPR-Cas9, including lower efficiency, challenges in cell delivery due to its large size, and the requirement of a longer time to implement genetic changes [86, 87]. Additionally, it can introduce unwanted byproducts like indels, posing risks of side effects and complications [88, 70].

While the clinical safety of prime editors is still being evaluated, current applications have mainly focused on cancer modeling. Ely et al*.* utilized prime editing to model lung and pancreatic cancer. This system introduced clinically relevant mutations, such as KRAS mutations associated with drug resistance and TP53 mutations in pancreatic cancer [89]. Another group established in vivo tumor models by inducing the S45 deletion of cadherin-associated protein beta 1 (CTNNB1) in somatic cells, achieving effective editing rates of over 80% at the target site [90]. The establishment of efficient disease models facilitates basic research and enhances our understanding of disease pathology and mechanisms.

### Dual role of Cas12 and Cas13 as gene editing and diagnostic tools

In recent years, extensive research has been conducted on the Cas12 and Cas13 systems, complementing the well-established Cas9 system. Cas12 has demonstrated high specificity in recognizing and cleaving double-stranded DNA [91, 92]. In contrast, Cas13 is distinguished by its unique recognition and targeted cleavage of single-stranded RNA [93]. Both Cas12 and Cas13 possess the characteristic of collateral cleavage, leading to unintended cleavage of non-targeted sequences [94, 95]. While this feature may not be ideal for therapeutic editing, it proves incredibly valuable in the development of CRISPR-based diagnostic tools. Consequently, several diagnostic approaches have been devised utilizing the Cas12a and Cas13a systems [23, 24].

### DNA editing mechanism of CRISPR-Cas12

**Fig. 3:**
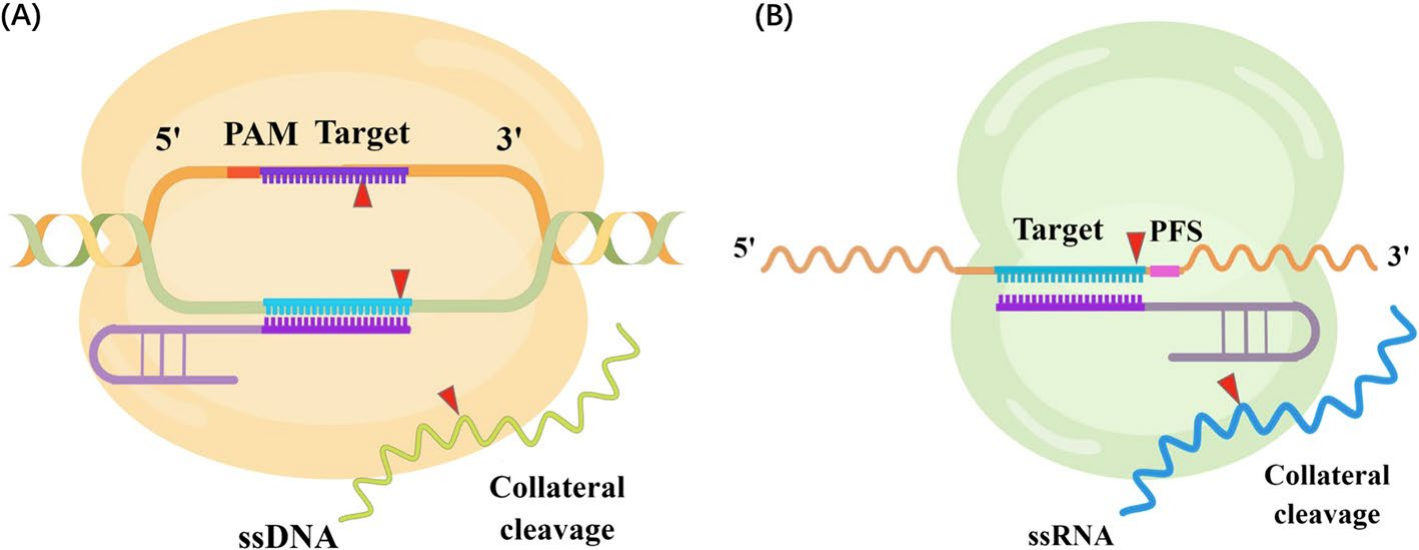
Schematic of CRISPR-Cas12a and CRISPR-Cas13a functions. **A** Cas12a nuclease recognizes DNA targets complementary to the crRNA spacer. Target recognition results in cleavage activities which leads to a staggering DNA double-strand break. In addition, activated Cas12a could nonspecifically cleave nearby ssDNA. **B** Cas13a is a crRNA-guided RNA-targeting nuclease. Upon binding to the target RNA, it could perform the target cleavage downstream of the protospacer flanking site (PFS, pink). In addition, activated Cas13a also possesses a nonspecific ribonuclease activity. The cleavage sites are marked by red arrows

Cas12 protein shares similar functions with Cas9 in recognizing and editing double-stranded DNA, but it relies solely on CRISPR RNA (crRNA) for guidance. Unlike the Cas9 system, which requires the host endonuclease RNase III for processing pre-crRNA into mature crRNA, Cas12 can independently carry out this step [96]. The Cas12 family can be further classified into Cas12a-i, but only Cas12a, b, f, and g have demonstrated potential in disease diagnosis [94]. Cas12a, the first identified type V CRISPR protein, consists of the REC and NUC lobes, which are further divided into the REC1 and REC2 domains, the RuvC, PAM-interacting (PI), and the Wedge (WED) domains, respectively (Fig. 1B) [97, 98]. The RuvC domain houses a single catalytic site responsible for the DNase activity [99], enabling sequential cleavage of the double-stranded DNA, with the non-target strand being cleaved first. This would result in a staggered-end DNA break with 5’ overhangs (Fig. 3A) [97].

Previous knowledge on Cas9 has emphasized the significance of the PAM region, which is also crucial for Cas12a. The PI, REC1, and WED domains of Cas12a enzymes collectively facilitate PAM recognition [100]. Cas12a typically recognizes a 5′-TTTV-3′ PAM [101, 102]. In addition, it exhibits a more flexible recognition capability, allowing for suboptimal PAM binding [103], such as 5′-TCTA-3′, 5′-TCCA-3′, and 5′-CCCA-3′. Under these circumstances, it retains the cleavage activity but at a lower efficiency [104]. Similar to Cas9, various Cas12a variants have been engineered to recognize alternative PAM sequences without compromising efficacy, broadening their range of applications [105].

### Application of the Cas12a in cancer therapy via target gene modification

Extensive investigations have been carried out to explore the application of Cas12a in human cells for genetic editing and diagnostics. Studies evaluating various Cas12a homologs have demonstrated their effective genome editing capabilities. In comparison to the well-established Cas9 editing system, Cas12a has exhibited slightly reduced efficiency but improved accuracy in editing [92]. This can be attributed to the longer spacer region of the CRISPR RNA (crRNA), which enhances specificity. Notably, the development of dead Cas12a has revealed its potential for target gene repression, surpassing the performance of the dCas9 system [106]. Another advantageous feature of the Cas12a system is its ability to autonomously process pre-mature crRNA, facilitating simultaneous multiplex gene regulation when multiple crRNAs are encoded on the plasmid [107].

Cas12a has emerged as a powerful tool for genome editing that could be applied in cancer therapy. Specifically, it has been designed to disrupt commonly mutated genes such as vrafmurine sarcoma viral oncogene homolog B (BRAF)-V600E [108, 109]. In addition, Choi et al*.* leveraged the multiplex gene targeting capability of Cas12a by designing systems that target three frequently mutated genes (TP53, Adenomatous polyposis coli (APC), and Phosphatidylinositol-4,5-bisphosphate 3-kinase catalytic subunit alpha gene (PIK3CA)), in colorectal cancer patients [110]. These studies have highlighted the unique advantages of Cas12a in therapeutic editing for cancer cells.

### Application of the Cas12a in cancer diagnosis utilizing collateral cleavage activity

Apart from its potential in gene therapy, Cas12a is widely employed as a diagnostic tool. This is accomplished through its collateral cleavage activity upon binding to the target. Upon binding, Cas12a undergoes a conformational change in the lid region, exposing the catalytic residue and enabling indiscriminate cleavage of single-stranded DNA (ssDNA) [111]. This collateral cleavage activity is a multiple turnover process, rendering it highly sensitive for detection purposes. Commonly mutated genes, such as TP53, Breast Cancer gene 1 (BRCA1), and KRAS, serve as popular tumor biomarkers [112, 113]. Cas12a has achieved attomolar sensitivity in detecting specific targets [23], and further studies have shown its superior sensitivity compared to droplet digital PCR (ddPCR) while offering the advantage of faster detection [114].

These features underscore the feasibility of Cas12a as an excellent diagnostic tool. Ongoing clinical trials primarily focus on genetic diseases or infections, with a single trial aiming to test the detection of Epstein-Barr virus (EBV) DNA in nasopharyngeal brushing and patient plasma as an indicator for nasopharyngeal carcinoma (NCT05447169) (Table 1). Further trials should be conducted to evaluate the effectiveness, safety, and feasibility of implementing Cas12a in a clinical setting. Additionally, expanding the range of target genes and diseases for therapeutic editing could provide new avenues for exploration.

### RNA editing mechanism of CRISPR-Cas13

Cas13 is a distinct CRISPR effector that operates through crRNA-guided RNA targeting and does not share structural homology with the other types of CRISPR proteins [115]. It consists of the REC and NUC lobes, and its target RNA cleavage is mediated by the two higher eukaryotes and prokaryotes nucleotide-binding (HEPN) domains (Fig. 1C) [115]. Cas13 can be categorized into four subtypes, including Cas13a, b1, b2, c, d, X, and Y [116]. Among these subtypes, Cas13a was the first to be discovered and extensively studied for its potential in gene editing and diagnostics. As an RNA-targeting nuclease, Cas13a interacts with the protospacer flanking site (PFS) instead of PAM recognition [117]. Notably, while bacterial cells have exhibited collateral cleavage of random single-stranded RNAs (ssRNAs) by Cas13a [95], this activity has not been observed in eukaryotic cells [93].

### Application of the Cas13a in cancer therapy via post-transcriptional regulation

Cas13a, a crRNA-guided RNA-targeting CRISPR effector, displays structural dissimilarity to other CRISPR proteins [115]. It exhibits diverse mechanisms for regulating gene expression, including gene knockdown [118], transcript tracking [93], and RNA editing [119, 120]. Previous studies have demonstrated its post-transcriptional knockdown ability on endogenous genes such as KRAS, C-X-C Motif Chemokine Receptor 4 (CXCR4), and Peptidylprolyl Isomerase B (PPIB) [93]. This form of regulation avoids direct alterations to genomic DNA, leading to more controlled outcomes. Other studies proved the potential of Cas13a gene therapy in lung cancer and chronic myelogenous leukemia [121, 122]. Recently, high-fidelity Cas13 variants, such as Cas13d and Cas13X, have been proposed to further enhance their therapeutic potential [123]. Compared to RNA interference (RNAi) systems, CRISPR-Cas13 exhibits improved specificity and efficacy, achieving over 90% knockdown efficiency. Moreover, off-target effects observed in RNAi systems are substantially reduced with CRISPR-Cas13 [124]. Both processes are highly efficient, with significant post-transcriptional suppression observed within a single day. However, the clinical application of the CRISPR-Cas13 system is hindered by challenges associated with in vivo delivery, limiting its potential in clinical settings [125].

Abudayyeh et al*.* proposed a modified version of Cas13a in which the catalytic activity is rendered inactive by mutating the arginine residues in the HEPN domains [93]. This modified Cas13a functions in a similar way to dCas9. It can be directed to specific targets but loses its editing capability. This expanded functionality allows for its application not only in transcriptome analysis but also in therapeutic approaches. However, despite these advancements, none of these approaches have progressed from the laboratory to clinical settings, emphasizing the need for more clinical studies to validate their potential in real-world applications.

### Application of the Cas13a in cancer diagnosis utilizing collateral cleavage activity

Cas13a exhibits collateral cleavage of adjacent non-target ssRNAs, enabling the design of diagnostic tools similar to Cas12a (Fig. 3B). When the crRNA-target RNA duplex binds to the NUC lobe of Cas13a, a conformational change occurs, activating its catalytic function. As the HEPN domains are exposed on the surface, free ssRNAs in the surrounding solution can be readily accessed and cleaved, resulting in collateral cleavage activity [126]. One notable example of such a diagnostic tool is the specific high-sensitivity enzymatic reporter unlocking (SHERLOCK) system. In this system, the target sequence is first amplified using techniques such as recombinase polymerase amplification (RPA) or reverse transcriptase (RT)-RPA to generate target DNA sequences. Subsequently, T7 transcription is employed to transcribe the target sequence into RNA. The resulting RNA sample then activates the Cas13a-gRNA complex, leading to the cleavage of fluorophore quencher labeled (FQ-labeled) ssRNA reporters. The intensity of the fluorescence signal can be used as a reference to determine the initial target concentration [24]

Previous studies have demonstrated the attomolar sensitivity of Cas13a in detecting low-frequency cancer mutations in cell-free DNA fragments (cfDNA), comparable to the sensitivity by ddPCR and quantitative PCR (qPCR) [127]. Gootenberg et al*.* successfully detected common mutations, such as Epidermal growth factor receptor (EGFR) L858R, T790M and BRAF V600E, in samples from patients with non-small cell lung cancer (NSCLC) [24]. More recently, researchers found Cas13a could also detect mRNA, miRNAs and even exosomal protein markers. Similar to cfDNA, mRNA of mutated genes, such as EGFR mRNA, could serve as an effective biomarker for NSCLC [128]. miRNAs have garnered significant attention in cancer biomarker research, and studies have demonstrated the ability of the Cas13a system to detect specific miRNA biomarkers. For example, miR-19b has been identified as a potential marker for medulloblastoma [129], while miR-17 and miR-21 have been identified as potential markers for breast cancer [130]. Other than its sensitivity, the specificity of Cas13a has been investigated. It has demonstrated the capability to distinguish between miR-17, miR-106a, miR-20a, and miR-20b, proving a single-nucleotide resolution [131].

Furthermore, research groups have designed more complexed circuits that allow Cas13a to detect protein markers, such as Programmed cell death 1 ligand 1 (PD-L1), Interleukin-6 (IL-6), and Vascular endothelial growth factor (VEGF), in patient serum [132, 133]. The system involved a target-specific aptamer, which would be amplified upon binding to the target. The amplified aptamer sequence can be transcribed by T7 polymerase, which simultaneously activates the Cas13a-crRNA complex and triggers its collateral cleavage activity [132]. These recent developments expand the scope of Cas13a-based detection and present promising potential for these tools to enter clinical trials.

### Benefits of Cas-based detection methods over conventional techniques

Cas-based detection methods offer several advantages compared to conventional detection methods such as real-time PCR (RT-PCR) or qPCR. RT-PCR and qPCR are labor-intensive, prone to human error, time-consuming, and expensive [134]. They require specialized equipment and lab environments. In contrast, CRISPR-based detections are portable, and commercial lateral flow strips enable rapid detection with results available within two hours [135]. The high sensitivity of Cas-based methods allows for single-copy viral detection, which is a significant advantage [23, 24]. Moreover, they are cost-friendly, particularly when combined with newly developed diagnostic tools [136].

In 2020, the introduction of Combinatorial Arrayed Reactions for Multiplexed Evaluation of Nucleic acids (CARMEN) further expanded the detection range and efficiency of the SHERLOCK by incorporating numerous optimized crRNAs [137]. CARMEN can accommodate more than 1000 samples or detect 169 pathogens for five samples in each chip, reducing the cost of SHERLOCK by 300-fold. Other groups have proposed innovative designs that combine CRISPR-Cas13 with microfluidic electrochemical biosensors, creating simple, easily scalable, and amplification-free diagnostic tools [129]. These advantages have generated significant interest in CRISPR-based diagnostic tools for infectious diseases and cancers with specific biomarkers.

## Conclusion

Over the years, the landscape of cancer treatment has witnessed remarkable progress, transitioning from traditional interventions to more innovative approaches such as immunotherapy and gene therapy [138]. Traditional cancer treatments have long been the mainstays of cancer management. However, updated approaches such as immunotherapy, which harnesses the body’s immune system to target and eliminate cancer cells, has revolutionized the field [139]. Additionally, gene therapies, including the use of CRISPR-Cas9 technology, hold promise for targeted genetic modifications and precision medicine.

The past decade has witnessed significant advancements in the understanding and application of CRISPR systems. Their simplicity, robustness, and high efficiency have opened up possibilities for the treatment and diagnosis of various diseases, including cancer [28]. This review has provided an overview of the structural and functional characteristics of Cas9, Cas12, and Cas13, as well as their applications in gene therapy and molecular diagnosis.

Despite the rapid development of CRISPR, which provides new hope for once-believed incurable diseases, there are still significant hurdles that limit its widespread application. These challenges include unstable editing efficiency, off-target editing concerns, and limitations in vivo delivery.

## Data Availability

Not applicable.

## Abbreviations

CRISPR: Clustered regularly interspaced short palindromic repeats
Cas: CRISPR-associated protein
DNA: Deoxyribonucleic acid
RNA: Ribonucleic acid
pre-crRNAs: Precursor-CRISPR RNAs
crRNA: CRISPR RNA
tracrRNA: Trans-activating crRNA
ssRNA: Single-stranded RNA
gRNA: Guide RNA
REC: Recognition lobe
NUC: Nuclease lobe
CTD: C-terminal domain
PAM: Protospacer Adjacent Motif
DSB: Double-stranded break
HDR: Homology-directed repair
NHEJ: Nonhomologous end joining
AAV: Adeno-associated virus
TP53: Tumor Protein P53
LKB1: Liver kinase B1
KRAS: Kirsten ratsarcoma viral oncogene homolog
KO: Knockout
ARID1A: AT-rich interaction domain 1A
CDKN2A: Cyclin-dependent kinase inhibitor 2A
TP53/CDKN2A(KO): TP53 and cyclin-dependent kinase inhibitor 2A (CDKN2A) knockout
GEJ: Gastro-esophageal junction
GeCKO: Genome-scale CRISPR-Cas9 knockout
PHGDH: Phosphoglycerate dehydrogenase
HCC: Hepatocellular carcinoma
CAR: Chimeric antigen receptors
MHC: Major histocompatibility complex
TCRβ: T-cell receptor β
PD-1: Programmed cell death protein 1
CTLA-4: Cytotoxic T lymphocyte-associated antigen-4
GVHD: Graft-versus-host disease
TCRα: T-cell receptor α
NY-ESO-1: New York esophageal squamous cell carcinoma 1
TRAC: T-cell receptor alpha constant
TRBC: T-cell receptor beta constant
neoTCR: Neoantigen-specific T-cell receptor
LOH: Losses of heterozygosity
BEs: Base editors
CBEs: Cytosine base editors
ABEs: Adenine base editors
PEs: Prime editors
APOBEC1: Apolipoprotein B MRNA Editing Enzyme Catalytic Subunit 1
dCas9: Dead CRISPR-associated protein 9
CGBE: C-to-G base editors
tBE: Transformer base editing
dCDI: Deoxycytidine deaminase inhibitor
PAK4/ERK: P21-activated Kinase 4/Extracellular Regulated Protein Kinases
RSK2/TP53BP1/γ-H2AX: Ribosomal S6 Kinase 2/Recombinant Tumor Protein p53 Binding Protein 1/Phosphorylated H2A Histone family member X
7CAR8: CD7-directed allogeneic CART
T-ALL: T-cell acute lymphoblastic leukemia
RT: Reverse transcriptase
pegRNA: Prime-editing guide RNA
CTNNB1: Cadherin-associated protein beta 1
CRISPRa: CRISPR activation
CRISPRi: CRISPR interference
XIST: X-inactive specific transcript
FOXP3: Forkhead box P3
PI: PAM-interacting
WED: Wedge
BRAF: Vrafmurine sarcoma viral oncegene homolog B
APC: Adenomatous polyposis coli
PIK3CA: Phosphatidylinositol-4,5-bisphosphate 3-kinase catalytic subunit alpha gene
BRCA1: Breast Cancer gene 1
ddPCR: Droplet digital polymerase chain reaction
EBV: Epstein-Barr virus
HEPN: Higher eukaryotes and prokaryotes nucleotide-binding
PFS: Protospacer flanking site
CXCR4: C-X-C Motif Chemokine Receptor 4
PPIB: Peptidylprolyl Isomerase B
RNAi: RNA interference
SHERLOCK: Specific high-sensitivity enzymatic reporter unlocking
RPA: Recombinase Polymerase Amplification
RT-RPA: Reverse transcriptase-RPA
FQ-labeled: Fluorophore quencher labeled
cfDNA: Cell-free DNA fragments
NSCLC: Non-small cell lung cancer
PD-L1: Programmed cell death 1 ligand 1
IL-6: Interleukin-6
VEGF: Vascular endothelial growth factor
RT-PCR: Real-time polymerase chain reaction
qPCR: Quantitative polymerase chain reaction
CARMEN: Combinatorial arrayed reactions for multiplexed evaluation of nucleic acids
NGS: Next-generation sequencing
FLASH: Finding low abundance sequences by hybridization
